# Mobile health treatment support intervention for HIV and tuberculosis in Mozambique: Perspectives of patients and healthcare workers

**DOI:** 10.1371/journal.pone.0176051

**Published:** 2017-04-18

**Authors:** José António Nhavoto, Åke Grönlund, Gunnar O. Klein

**Affiliations:** 1 Informatics, School of Business, Örebro University, Örebro, Sweden; 2 Informatics, Department of Mathematics and Informatics, Eduardo Mondlane University, Maputo, Mozambique; Agencia de Salut Publica de Barcelona, SPAIN

## Abstract

**Background:**

Studies have been conducted in developing countries using SMS to communicate with patients to reduce the number of missed appointments and improve retention in treatment, however; very few have been scaled up. One possible reason for this could be that patients or staff are dissatisfied with the method in some way. This paper reports a study of patients’ and healthcare workers’ (HCW) views on an mHealth intervention aiming to support retention in antiretroviral therapy (ART) and tuberculosis (TB) treatment in Mozambique.

**Methods:**

The study was conducted at five healthcare centres in Mozambique. Automated SMS health promotions and reminders were sent to patients in a RCT. A total of 141 patients and 40 HCWs were interviewed. Respondents rated usefulness, perceived benefits, ease of use, satisfaction, and risks of the SMS system using a Likert scale questionnaire. A semi-structured interview guide was followed. Interviews were transcribed and thematic analysis was conducted.

**Results:**

Both patients and HCW found the SMS system useful and reliable. Most highly rated positive effects were reducing the number of failures to collect medication and avoiding missing appointments. Patients’ confidence in the system was high. Most perceived the system to improve communication between health-care provider and patient and assist in education and motivation. The automatic recognition of questions from patients and the provision of appropriate answers (a unique feature of this system) was especially appreciated. A majority would recommend the system to other patients or healthcare centres. Risks also were mentioned, mostly by HCW, of unintentional disclosure of health status in cases where patients use shared phones.

**Conclusions:**

The results suggest that SMS technology for HIV and TB should be used to transmit reminders for appointments, medications, motivational texts, and health education to increase retention in care. Measures must be taken to reduce risks of privacy intrusion, but these are not a main obstacle for scaling up systems of this kind.

## Introduction

The rapid growth of mobile technologies has led to the development of mobile health (mHealth) interventions aiming at helping treat or avoid a range of health conditions. Tuberculosis (TB) and HIV/AIDS are two major public health concerns. Alone, TB and HIV are leading causes of infectious disease deaths; together, TB causes one-third of AIDS-related deaths every year [1]. Mozambique is one of the countries in the world that are most burdened by HIV and TB. In 2015, Mozambique had 1.5 million people living with HIV/AIDS (PLHIV) and 223 new infections and 108 deaths related to HIV occur per day [2]. In Maputo Province and Maputo City, the adult HIV prevalence rate was 19.8% and 16.8% respectively (2009) [3]. In 2015, the estimated incidence rate of TB was 551 per 100,000 population [4]. While treatment has improved over the years, an important remaining problem is to make patients stay in treatment programs. Interventions to improve adherence to antiretroviral therapy (ART) and TB treatment are urgently needed in HIV-infected patients, particularly in developing countries where the majority of PLHIV reside [5].

One of the strategies for improving adherence includes communication between the healthcare provider and patient using short message service (SMS), as this medium is comparatively cheap and can reach many people as mobile phones are common in most countries [6]. Such communication can be used for several purposes, such as facilitating timely access to relevant and supporting health information, making the doctor-patient communication easier, providing context-specific support, reminding of medication and treatment [7–9], and providing educational and motivational messages [7,9]. In general, efforts addressing several dimensions of adherence have been found more effective than single target interventions [10].

Text messaging is in common use, but results are not consistent[11,12]. Nhavoto and Grönlund [13] surveyed 220 studies that investigated this issue among patients undergoing treatment for different clinical conditions such as HIV/AIDS, hypertension, and asthma. Most studies showed benefits from electronic reminders in the short term, but these results should be viewed with caution since most are pilot and small-scale trials [14,15] and done in different ways.

The fact that there are so many variations of intervention design in SMS text messaging trials makes it unclear what specific ingredients actively and successfully promote adherence. There are some studies that try to understand users’ perceptions of mHealth intervention. A study in India of an intervention comprising an automated interactive voice response call and a pictorial SMS, each delivered weekly, found that participants perceived the intervention as a sign of “care” from the clinic [16]. However, they also perceived a risk of unintentional disclosure of their HIV status and the resulting stigma. Studying patient and healthcare workers (HCW) views of mHealth interventions helps to understand why an intervention works or not. Therefore, this study investigates perspectives of patients and HCWs regarding SMS use in a randomised control trial (RCT) aiming at improving patient retention in HIV and TB-HIV care. We also investigate if and how demographics affect patients’ attitudes towards the SMS communication.

## Methods

### Study context

This study draws on a survey within the SMSaúde trial, an RCT that studied the effect of mobile phone SMS reminders on adherence to ART [17] and TB treatment in the Maputo province of Mozambique. There were five recruitment sites. Patients on ART were recruited at the healthcare centres Machava II, Matola II, and Namaacha, and TB patients at Machava II, Matola I, Matola II, and Ndlavela. All healthcare centres provide intense ART and TB care. Newly diagnosed TB patients receive six months of standard TB chemotherapy in two phases: an intensive phase for two months during which they get treatment every day and a continuation phase of four months during which they visit the healthcare centre almost every week. Patients collect anti-TB drugs daily during the intensive phase (except weekends) and weekly or monthly during the continuation phase. Voluntary counselling and testing for HIV is offered to all TB patients. Patients on ART collect antiretroviral drugs monthly and generally have appointments monthly.

### Interventions

Between 2012 and 2015 two RCTs were implemented. The first tested an mHealth ART retention support intervention in three healthcare centres, and the second tested a similar intervention for retention support of HIV-infected TB patients in four centres. While the HIV treatment is generally lifelong, the TB patients received six to eight months of treatment. In the SMSaúde RCT, patients were eligible for participation if they were over 18 years old, able to access a mobile phone on a near-daily basis, and communicate via SMS text message. People who did not own mobile phones were eligible if they had shared access (with corroborative agreement by the phone owner). TB patients were eligible if they were initiating TB treatment for the first time.

Both interventions consisted of a structured series of SMS text messages sent automatically. The design, development, and implementation of both interventions as well the frequency and examples of the content of SMS have been reported elsewhere [17,18]. Patients received automated SMS text messages sent based on their medical appointments and drug pick-ups. Also, patients on TB treatment received motivational messages, and both PLHIV and TB patients received educational messages. SMS reminders were sent seven and two days before their scheduled appointment and/or drug pick-ups. Participants on ART care received SMS messages from March 2012 to September 2014, and those on TB care received SMS messages from October 2013 to March 2015.

### Participants

The survey was conducted as telephone interviews with patients and on-site interviews with HCWs. HCWs included medical practitioners (medical doctor, clinical technician, nurse, and health counsellor), pharmacy technicians and data-entry clerks.

**Fig 1:**
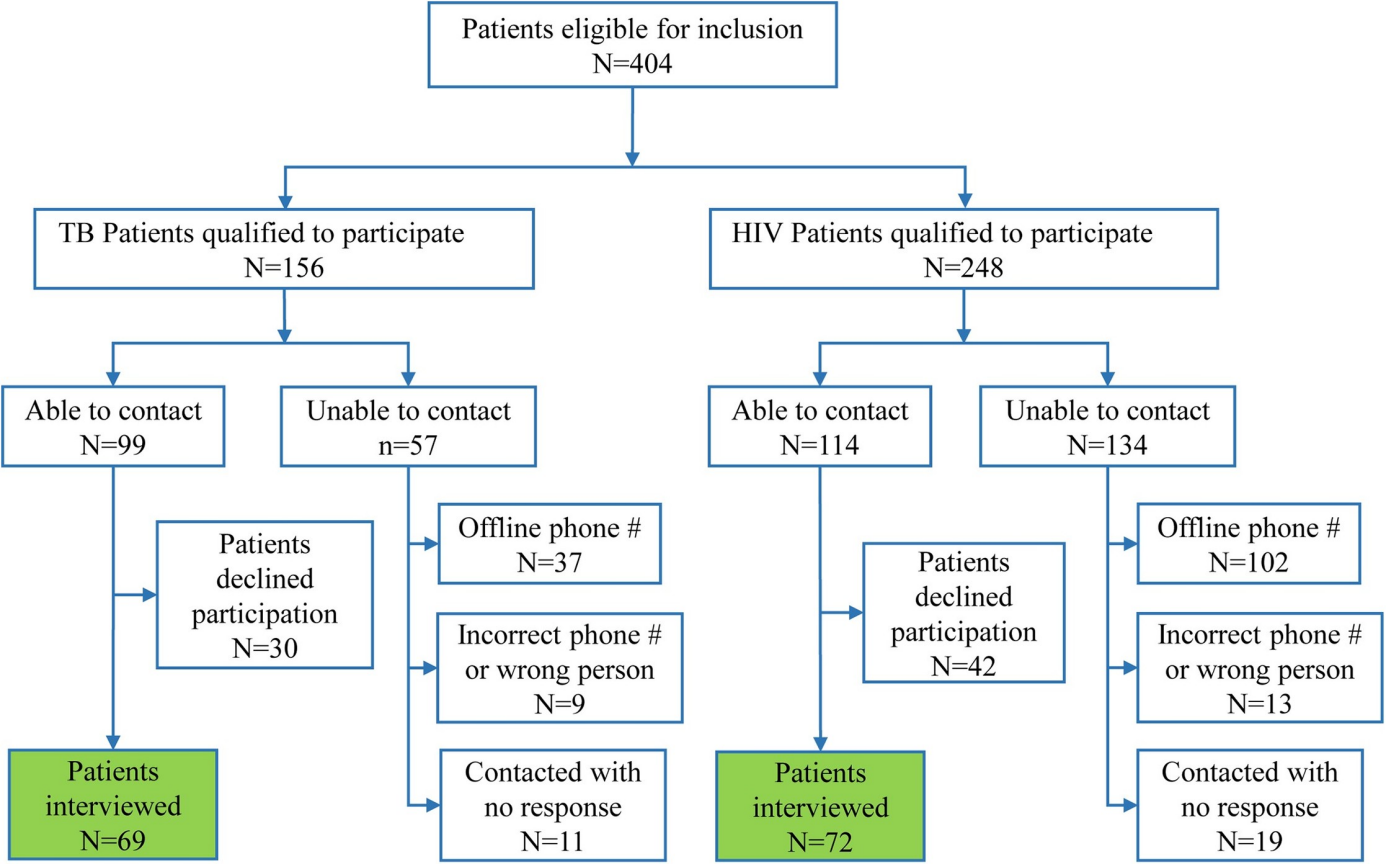
Participants interview enrolment flow chart.

Patients selected for the present study were from the intervention arm, that is, those who received SMS. In general, participants were eligible for this study if they (a) participated in the SMSaúde trial on ART treatment and received SMS messages or (b) participated in the SMSaúde trial in TB treatment and received SMS messages. For TB, we searched in the database for patients who had abandoned treatment, were cured, or had completed treatment. For PLHIV, we searched for those who had abandoned the treatment or were still in treatment. The combined database search yielded 404 eligible participants (156 TB patients and 248 PLHIV). Fig 1 summarises the details of the enrolment process. Of the 404 eligible patients, 213 could be contacted. Of the 213 patients contacted, 66% (141 individuals) agreed and were interviewed; this was 35% of the total number of patients.

The inclusion criterion for HCWs was to be from a health centre that participated in the SMSaúde study and provided health services to patients with HIV and/or TB. All the 40 HCWs in the five centres were included, and all agreed to be interviewed.

Among the HCWs, a majority were female (65%). They were aged 25–53 years (median age: 34). Medical practitioners represented 70% of all HCWs. Only the data-entry clerks were technically involved with the SMS system.

One-hundred-forty-one patients participated, 69 with TB and 72 with HIV (S1 Table). A majority were female (63%) and in a relationship (52%), with a median age of 38 years (IQR 34–45 years). Half of the participants (51%) reported having children in their household. A majority (68%) were employed or self-employed, and common employments included housekeeper and merchant. A majority (66%) of participants had an average monthly household income lower than 156 USD. A large majority of participants (71%) used public transportation to go to their healthcare centre, and most of them spent between 5 to 29 minutes on the trip, but 20% spent more than one hour. A majority (52%) of the participants spent at least 6.25 USD monthly on transportation to visit the healthcare centre. Sixty-six percent had a monthly household income ≤ 156 USD.

### Data collection

The interview guide for the patients and the questionnaire for the HCW were developed and pilot tested before data collection. All telephone interviews were conducted in a quiet room and audio-recorded with participants’ consent. Interviews were conducted in Portuguese and took approximately 30 minutes. The questionnaire for HCWs comprised 13 questions (S1 Appendix). The patient interviews comprised 27 questions that included socio-demographic characteristics, socio-economic characteristics, therapy or drug-related information, social and SMS system-related variables (S2 Appendix). The SMS system-related variables included questions that explored participant perceptions of the system, and these included helpfulness of the intervention, ease of use, risks involved with the intervention, benefits, and their intention to reuse the system.

### Data analysis

Two health experts conducted the interviews in Portuguese and transcribed the recordings. The first author checked the transcriptions to ensure consistency with the recordings and translated the transcriptions into English. For qualitative analysis, data were coded and underwent thematic content analysis [19]. Coding and analysis were iterative, and themes were derived by frequency of codes (most occurring concepts) and repetition of perceptions across participants. For statistical analysis, basic descriptive statistics assessed frequency, percentages, median, and interquartile ranges. Man-Witney U test, Fisher’s exact test, and Spearman’s Rho correlation test were used to understand relationships between patients’ and HCWs’ perspectives.

### Ethical approval

The study protocol, field instruments, and informed consent forms were approved from an ethical perspective by the Regional Ethical Review Board at Uppsala, Sweden (Dnr 2105/082) and the Institutional Bioethics Committee on Health, Faculty of Medicine of Maputo Central Hospital (CIBS FM&HCM), Maputo, Mozambique (study code CIBS FM&HCM/79/2014). All participants provided written or verbal informed consent in Portuguese before their enrolment.

## Results

Patients reported the number of times they had missed appointments, the number of times they had missed collecting their medication, and the reasons for that. The majority of PLHIV (90%, 61/68) and the majority of TB patients (88%, 60/68) reported not having missed appointments. Out of those who missed, most reported that this had happened on 1–5 occasions. As the survey was anonymous, we could not check these reports directly, but the numbers are in alignment with the retention rates we could measure in the patient database, which were 85% and 87% respectively.

Patients reported missing their appointments because they had travelled, were working, were admitted to hospital, were not feeling well, or were told to go to the healthcare centre the next day.

A majority of the patients (HIV 82% [56/68], TB 97% [65/67]) reported not having missed collecting their medication at any time. This is consistent with the rate of collection of medication recorded in the patient database (78% and 94% respectively). Despite receiving SMS reminders, the most common reason for not collecting medication was forgetfulness. Other reasons included allergic reactions to medication, feeling better and not being able to go to the health centre because of unexpected events.

### Perceptions of participants towards the SMS system

#### Healthcare workers’ perceptions

Almost all HCWs (95%) agreed or strongly agreed with the statement, ‘The SMS system helped to reduce the number of patients who missed appointments’ (none disagreed). All HCWs agreed or strongly agreed that ‘The SMS system helped to reduce the number of patients who missed collecting medications’. A large majority of the HCWs (83%) agreed or strongly agreed with the statement, ‘The SMS system helped to discuss health-related issues with patients’. Only 13% participants were neutral, and the remaining two people disagreed or strongly disagreed.

Ninety-five percent of the HCW strongly agreed or agreed with the statement ‘There are benefits of motivational and/or educational messages’.

To the statement, ‘I think there are risks with the SMS system’, a majority (58%) agreed or strongly agreed. 28% disagreed. The main risk mentioned was unintentional disclosure of individuals’ HIV status. Participants were concerned that unauthorised access to text messages could cause problems for patients, especially in the context where shared phones are used to receive reminders (some patients used mobile phones of their relatives). Participants feared that the disclosure of patients’ HIV-status could motivate patients to abandon the treatment.

Text messages can be read by a family member or spouse who doesn’t know that the patient is HIV positive and is taking medication. (Clinical technician)

There are cases of patients with HIV who don’t disclose their health status to their relatives. Some relatives get surprised and share the information with others when they get the text messages. There is a risk of the patient giving up the treatment. (Health counsellor)

Medical practitioners were also concerned with the fact that patients could lose their phone (or have it stolen) and someone else could then learn that the phone owner had a particular health condition, in the event the person who found/stole the phone knew the patient.

Pharmacy and clerical technicians raised the concern that some partners of patients could mistrust their spouse because of receiving regular messages. In some cases, partners are not aware of the HIV status and those infected have chosen not to disclose it. Such a situation might create misunderstandings between spouses, or worse.

All HCWs agreed or strongly agreed with the statement, ‘I would recommend other healthcare centres to use the SMS system’. As for the reasons reported, the following four themes emerged.

**Perceived adherence and compliance to treatment:** Participants thought that the SMS system helps patients to stay in treatment and increases treatment compliance. Also, the system reduces the number of dropouts and the number of patients lost to follow-up.

**Perceived adherence to medication:** Participants thought that the system improves adherence to medication, reminding patients to collect medication on time. Also, SMS reminders help patients not only to collect medication but also to comply with dosage instructions.

**Perceived adherence to medical appointments:** Participants thought that the SMS system improves adherence to medical appointments.

**Perceived improved education, communication, and usefulness:** Participants thought that the system improves patient awareness of his/her disease while also improving the communication between provider and patient. They also reported that patients express happiness when they receive SMS text messages.

#### Perceptions of patients

**Fig 2:**
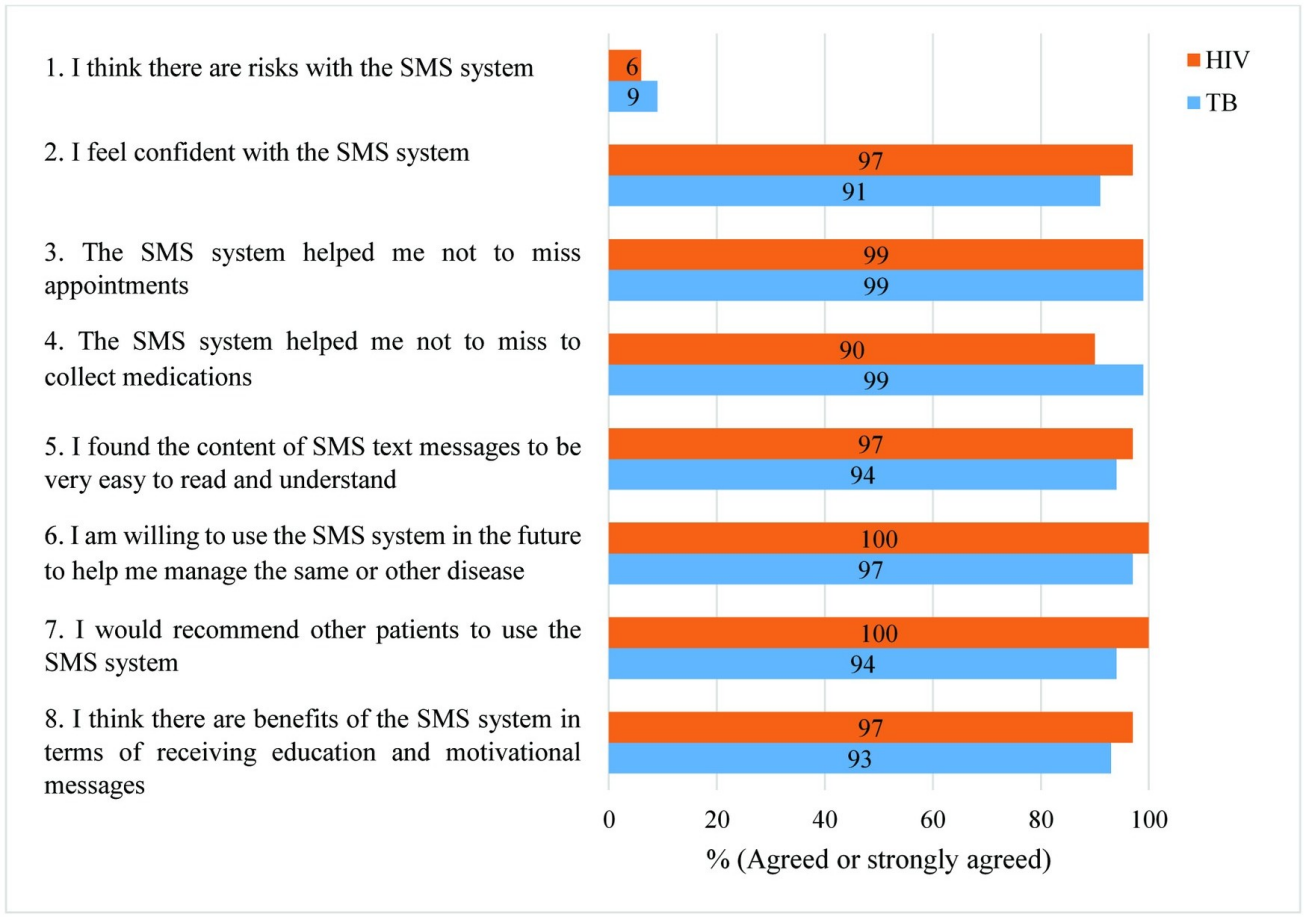
Summary of patient perceptions.

Fig 2 summarises patient perceptions (see also S2 Table).

In quite sharp contrast to the HCWs, a large majority of PLHIV (90%, 60/67) and TB patients (87%, 56/68) strongly disagreed or disagreed with the statement, ‘I think there are risks with the SMS system’. A vast majority of the patients were not concerned about risks with the systems, but those who did voice concerns were very concerned and mentioned, similar to the HCW, mainly the risk of disclosure of health status, stigma, and discrimination. Patients were concerned about the risk of unauthorised access to their mobile phone text messages.

If someone accesses my phone and sees the text messages from the hospital, then this person will know that I am HIV. (HIV-infected patient)

I am afraid because my sisters can access my phone without my consent and they can get to know my health status. (TB patient)

All PLHIV (67/67) and 94% (64/68) of the TB patients agreed or strongly agreed with the statement, ‘I would recommend other patients to use the SMS system’. Patients reported feeling support from their healthcare providers as a result of the interactive text messages (where they could also send messages and get a response). Patients felt motivated and appreciated that there was someone who cared about their health.

In responding to the question ‘How would you rate the ease of use of the SMS system’, patients used the Likert scale in which 1 = very difficult, 2 = difficult, 3 = neutral, 4 = easy, and 5 = very easy. A large majority of the TB patients (77%, 51/66) reported it to be easy or very easy to use the system. The PLHIV patients were less enthusiastic but not negative: 40% (27/68) thought it was easy or very easy to use, 54% were neutral.

#### Automatic SMS response

In contrast with the typical SMS reminder system, this one allowed for two-way communication. Patients could send text messages to the system using an automated SMS feature. The SMS system could recognise 15 different questions posed in free text by the patients. In response to each of these recognised questions, the system supplied a standard text answer. If the system could not understand the question, the reply would be, ‘Please contact the nearest healthcare centre’. The patients appreciated this feature very much. In response to the statement, ‘There are benefits of the SMSaúde system in terms of answering questions you have sent’, 53% of PLHIV and 83% of TB patients reported having perceived benefits or great benefits. None of the patients reported having received inappropriate SMS messages from the system. Afterwards, we manually examined all the questions typed by the patients and the automatic interpretation. There were no misinterpretations.

#### Perceptions of patients vs. demographics

Although previous research does not provide definitive guidance for how demographics might affect SMS use in this context, one might expect that people who live far from a healthcare centre would miss more appointments than those who live nearby and that women and people with families might be expected to care more about health issues than others. To test such ideas, we examined various demographic factors and found virtually no relevant significant correlations between them and (reported) outcomes, such as missing appointments. We also looked for potential differences between the two groups of patients and found no significant differences.

Neither age nor marital status, having children, one’s occupation, or distance from home to the healthcare centre were significantly correlated with differences in any of the perceptions of the group as a whole.

There was a significant correlation between the average income of PLHIV patients and agreement with the statement, ‘The SMS system helped me not to miss appointments’. Patients with an average monthly household income of less than 78 USD were less positive than those with an income between 78 and 156 USD (p = .039). They were also less in agreement with the statement, ‘How would you rate the ease of use (ease of interaction) of the SMS system’.

#### Reported content missing in the SMS system

Both patients and medical practitioners suggested some additions to the SMS system. Patients with TB advised that the system should include messages that provide information about diet and nutrition during the treatment, more messages that discuss how to prevent the disease, messages in local languages, and that healthcare providers should call once a month to check on the patient. Patients with HIV suggested that the system should provide more advice, explain more about the disease and advantages of taking medication, include messages that encourage voluntary testing of family members and more text messages about the importance of using a condom. Also, patients indicated they would like to receive SMS messages that are in capital letters and with information that can help one live a healthier life.

Medical practitioners advised that the system should include more educational and motivational messages, especially for PLHIV patients. In addition to SMS messages, they recommended including voice message reminders and improving patient confidentiality.

#### Recommendations about the SMS project

Pharmacy and clerical technicians suggested that the SMS project offer free mobile phones to patients who cannot afford one. They recommended that the SMS project partner with mobile-phone operators who could donate mobile phones and initial SIM cards, as some mobile operators provide cheap phones pre-loaded with SIM cards. HCWs recommend the SMS project be implemented nationwide to include more patients. They also recommend using the system as a model for communication with patients to help standardise information and ensure greater patient adherence to treatment. Medical practitioners expressed a wish to access messages sent by patients.

## Discussion and conclusions

This is the first study that provides a detailed description of patients’ and HCW’s views regarding the mobile-phone-based communication system in support of HIV and TB treatment in Mozambique. User views are important for researchers and for service providers in applying novel approaches to improve care.

The results suggest that patients and HCWs share positive views about the SMS system as a health promotion intervention for HIV and TB care. Patients were quite enthusiastic about the benefits they perceived in the mHealth system. Their comments suggest that SMS messages may encourage better healthcare for PLHIV and TB patients and may indeed be a useful addition to existing services. This is in line with Heron and Smyth’s literature review [20], which reported that mobile technology service users, when asked about their opinions, were happy with mobile health promotion interventions. In our study, however, patients with the lowest income were somewhat less positive. One interpretation suggests that poverty makes a difference; people with the lowest income may be less literate than others and more likely to share a phone. Also, the fact that some in the study suggested voice messages rather than text messages could indicate that literacy is an issue.

At the same time, participants—staff more so than patients—identified some risks associated with the SMS system. The main concern was possible confidentiality breaches with ensuing risk of stigmatisation and discrimination. In particular, they saw a risk of unintended disclosure of one’s HIV status if others were able to view text messages intended to be private. This was a real risk in some cases where patients share the phone with another family member. Despite their neutral content, text messages reminders about medication and appointments suggest that the patient is under treatment for some disease. The common practice in families in many communities, especially poor communities, of sharing a mobile phone could be the reason for this expression of concern. Although patients in the SMS trial were asked to provide contact numbers only after an agreement with the phone owner, some patients feared HIV-stigma resulting from unintended disclosure of their HIV status. The concern with confidentiality was not unexpected; similar concerns were found by Lester and colleagues [21] and by Curioso and colleagues [22]. The perceived risk of unintended disclosure of HIV status was also reported in studies in Cameroon [9], China [23], Kenya [24], Peru [22] and South Africa [25]. However, some studies have reported a preference for text messages over other forms of communication as they were considered to provide better confidentiality [16,26,27].

In the present study, self-reported adherence to appointments and medication was very good. These reports from patients and HCWs are consistent with the findings of our earlier evaluation study of the electronic health records [17] of this SMSaúde research project. This study found that the retention in antiretroviral therapy was excellent in both control and intervention group (91% vs. 94%). There was a significantly improved retention among urban patients (94% in the intervention group vs. 90% in the control group). This retention rate is much higher compared to the figure of 74% reported for Mozambique in 2010 and 2011 [28]. There may be many factors influencing these results. Generally, urban IT users are more prone to use information technologies than rural ones. They also have to travel shorter distances to visit the doctor and pick up their medicine. It may also be that the project served to improve care for all patients. The fact that the retention rate in our study was so high compared to earlier measurements also in the control group may be due to the generally increased focus on retention among HCWs in the project which may have affected all patients positively. The HCWs did not know which patients were in which group and would, of course, be interested in improving retention for all patients. Maybe they increased their efforts to convince patients to stay in the program. With this high retention rate of some 90%, it becomes hard to demonstrate significant increase with the intervention.

The higher self-reported adherence to medication in the TB group compared to the PLHIV group may have resulted from the different treatment regimens of the groups. While for PLHIV, medications are collected monthly, TB patients are due to collect medication daily in the first two months of their treatment and weekly for the remaining four months. It is less likely that patients forget or are late for something they have to do every day than something they do once a month. Put another way, the SMS system may be more useful for PLHIV where oblivion is a bigger risk.

Patients also reported appreciation of the concern of their healthcare providers for their health, as evident from the text messages. Reports of similar interventions providing support and making the recipient feel valued were also reported in Kenya [24,29,30] and India [16]. The feeling of being supported and cared about by their healthcare provider was also reported in a study from British Columbia [31] in a study that used text messages in a tuberculosis clinic.

The design of our SMS system allowed a measure of two-way communication, which seems to have enhanced engagement, improved patient-provider relationships, and increased trust. Similar findings are also reported in a study by Johnson et al. [32]. Evidence suggests that patients are better able to manage their care and are more likely to achieve adherence goals when they perceive their providers care about and like them [33,34]. It is possible that the messages back and forth evoked a relationship dynamic that further boosted the existing patient-provider relationship. Furthermore, the study by Hoffman and colleagues [35] found that SMS messaging helped patients feel less isolated and that they valued the reminders and welcomed messages regarding health-related issues. The fact that patients see benefits of interacting with the SMS system reveals that their interest is not only in receiving reminders but also in something that lifts their self-esteem and gives encouragement [22].

While this study provides significant insights into views of patients and healthcare workers and highlights strengths and weaknesses of mHealth interventions in Mozambique, there are some limitations. Firstly, the sample of patients was recruited within two RCTs where the HIV trial was completed a year earlier at the time of the interviews, which might have limited the reach of these patients. This may be the explanation for the higher dropout of HIV patients than for TB patients (71% vs 56%). Secondly, as there was quite high dropout for several reasons, including that people often change their phone number and sometimes don’t have their own phone but use someone else’s; it was only possible to interview 35% of those eligible, which is of course a limitation. There was, however, no systematic dropout and the views among those interviewed were very much in agreement.

### Conclusions

We examined patient and HCW perspectives on the use of SMS messaging to improve HIV and TB treatment adherence in Mozambique. Participants overall expressed very positive views, but concerns were raised about possible confidentiality breaches. It is important that patients are fully aware of such risks and may decline, especially if they do not own the telephone they use. The unique feature in this system with automatic recognition of questions from the patients and the provision of appropriate answers was much appreciated. We believe this is a feature of systems like the one studied here that should be considered and further developed in future implementations. For instance, the knowledge base could be extended so it could answer more questions. Self-reported outcomes are one way of measuring patients’ outcomes, but our experience also stresses the importance of evaluating effectiveness based on data in provider registers. Overall, this study shows support for the use of SMS technology to transmit reminders for appointments, medications, motivational texts, and health education information. Future interventions should also consider using multimedia messaging services (MMS), as also recommended by some participants, to further enrich patient-provider interactions. For example, MMS would help in communicating with people with low levels of literacy.

## Supporting information

S1 Appendix Questionnaire for health care workers. (DOCX)

S2 Appendix Interview guide for patients. (DOCX)

S1 Table Demographic characteristics of patients. (DOCX)

S2 Table Summary of patients’ responses. (DOCX)

## References

[1] World Health Organization. Uniting to end TB/HIV coinfection [Internet]. 2016 [cited 2016 Apr 6]. Available from: http://www.who.int/hiv/mediacentre/news/tb-hiv-day-2016/en/

[2] Conselho Nacional de Combate ao HIV e SIDA (CNCS). Resposta Global à SIDA, Relatório do Progresso, Moçambique [Internet]. Maputo, Mozambique; 2016 [cited 2016 Apr 6]. Available from: http://www.unaids.org/sites/default/files/country/documents/MOZ_narrative_report_2016.pdf

[3] Instituto Nacional de Saúde (INS). National Survey on Prevalence, Behavioral Risks, and Information about HIV and AIDS in Mozambique [Internet]. 2010 [cited 2016 Apr 6]. Available from: http://www.measuredhs.com/pubs/pdf/HF33/HF33y.pdf

[4] World Health Organization. Mozambique: Tuberculosis Profile [Internet]. 2015 [cited 2016 Apr 9]. Available from: https://extranet.who.int/sree/Reports?op=Replet&name=/WHO_HQ_Reports/G2/PROD/EXT/TBCountryProfile&ISO2=MZ&outtype=PDF

[5] DeSilva MB, Gifford AL, Keyi X, Li Z, Feng C, Brooks M, et al Feasibility and Acceptability of a Real-Time Adherence Device among HIV-Positive IDU Patients in China. AIDS Res Treat. 2013;2013:1–6. 10.1155/2013/957862 PMC3730150 23956851

[6] Shaw RJ, Bosworth HB, Hess JC, Silva SG, Lipkus IM, Davis LL, et al Development of a Theoretically Driven mHealth Text Messaging Application for Sustaining Recent Weight Loss. JMIR mHealth uHealth. 2013;1(1):1–14. 10.2196/mhealth.2343 PMC4114452 25100678

[7] Pop-Eleches C, Thirumurthy H, Habyarimana JP, Zivin JG, Goldstein MP, de Walque D, et al Mobile phone technologies improve adherence to antiretroviral treatment in a resource-limited setting: a randomized controlled trial of text message reminders. AIDS. 2011;25(6):825–34. doi: 10.1097/QAD.0b013e32834380c1 21252632 10.1097/QAD.0b013e32834380c1 PMC3718389

[8] Puccio JA, Belzer M, Olson J, Martinez M, Salata C, Tucker D, et al The use of cell phone reminder calls for assisting HIV-infected adolescents and young adults to adhere to highly active antiretroviral therapy: a pilot study. AIDS Patient Care STDS. 2006;20(6):438–44. doi: 10.1089/apc.2006.20.438 16789857 10.1089/apc.2006.20.438

[9] Mbuagbaw L, Thabane L, Ongolo-Zogo P, Lester RT, Mills EJ, Smieja M, et al The Cameroon Mobile Phone SMS (CAMPS) trial: a randomized trial of text messaging versus usual care for adherence to antiretroviral therapy. PLoS One. 2012;7(12):e46909 doi: 10.1371/journal.pone.0046909 23236345 10.1371/journal.pone.0046909 PMC3516507

[10] Roter DL, Hall JA, Merisca R, Nordstrom B, Cretin D, Svarstad B. Effectiveness of interventions to improve patient compliance: a meta-analysis. Med Care. 1998;36(8):1138–61. 9708588 10.1097/00005650-199808000-00004

[11] Haynes RB, Ackloo E, Sahota N, McDonald HP, Yao X. Interventions for enhancing medication adherence. Cochrane database Syst Rev. 2008;(2). 10.1002/14651858.CD000011.pub3 18425859

[12] Nieuwlaat R, Wilczynski N, Navarro T, Hobson N, Jeffery R, Keepanasseril A, et al Interventions for enhancing medication adherence. Cochrane Database Syst Rev. John Wiley & Sons, Ltd; 2014;(11). 10.1002/14651858.CD000011.pub4 PMC7263418 25412402

[13] Nhavoto JA, Grönlund A. Mobile technologies and geographic information systems to improve health care systems: a literature review. JMIR mHealth uHealth. 2014;2(2):e21 doi: 10.2196/mhealth.3216 25099368 10.2196/mhealth.3216 PMC4114429

[14] Anhøj J, Møldrup C. Feasibility of collecting diary data from asthma patients through mobile phones and SMS (short message service): response rate analysis and focus group evaluation from a pilot study. J Med Internet Res. 2004;6(4):e42 doi: 10.2196/jmir.6.4.e42 15631966 10.2196/jmir.6.4.e42 PMC1550628

[15] Earth Institute. Barriers and Gaps Affecting mHealth in Low and Middle Income Countries: A Policy White Paper. Washington, D.C.: mHealth Alliance; 2010. 1–79 p.

[16] Rodrigues R, Poongulali S, Balaji K, Atkins S, Ashorn P, De Costa A. “The phone reminder is important, but will others get to know about my illness?” Patient perceptions of an mHealth antiretroviral treatment support intervention in the HIVIND trial in South India. BMJ Open. 2015;5(11):e007574 doi: 10.1136/bmjopen-2015-007574 26525717 10.1136/bmjopen-2015-007574 PMC4636629

[17] Davey DJ, Nhavoto JA, Augusto O, Ponce W, Traca D, Nguimfack A, et al SMSaúde: Evaluating mobile phone text reminders to improve retention in HIV care for patients on antiretroviral therapy in Mozambique. JAIDS J Acquir Immune Defic Syndr. 2016;73(2):e23–30. doi: 10.1097/QAI.0000000000001115 27632147 10.1097/QAI.0000000000001115 PMC5767278

[18] Nhavoto JA, Grönlund Å, Chaquilla WP. SMSaúde: Design, Development, and Implementation of a Remote/Mobile Patient Management System to Improve Retention in Care for HIV/AIDS and Tuberculosis Patients. JMIR mHealth uHealth. 2015;3(1):e26 doi: 10.2196/mhealth.3854 25757551 10.2196/mhealth.3854 PMC4376127

[19] Braun V, Clarke V. Using thematic analysis in psychology. Qual Res Psychol. 2006;3:77–101.

[20] Heron KE, Smyth JM. Ecological momentary interventions: incorporating mobile technology into psychosocial and health behaviour treatments. Br J Health Psychol. 2010 2;15(Pt 1):1–39. doi: 10.1348/135910709X466063 19646331 10.1348/135910709X466063 PMC2800172

[21] Lester RT, Gelmon L, Plummer FA. Cell phones: tightening the communication gap in resource-limited antiretroviral programmes? AIDS. 2006;20(17):2242–4. doi: 10.1097/QAD.0b013e3280108508 17086071 10.1097/QAD.0b013e3280108508

[22] Curioso WH, Alex Quistberg D, Cabello R, Gozzer E, Garcia PJ, Holmes KK, et al “It’s time for your life”: How should we remind patients to take medicines using short text messages? AMIA Annu Symp Proc. 2009;2009:129–33. 21633523 PMC3105604

[23] Huang D, Sangthong R, McNeil E, Chongsuvivatwong V, Zheng W, Yang X, et al Effects of a Phone Call Intervention to Promote Adherence to Antiretroviral Therapy and Quality of Life of HIV/AIDS Patients in Baoshan, China: A Randomized Controlled Trial. AIDS Res Treat. Hindawi Publishing Corporation; 2013;2013. 10.1155/2013/580974 PMC3562599 23401755

[24] van der Kop ML, Karanja S, Thabane L, Marra C, Chung MH, Gelmon L, et al In-depth analysis of patient-clinician cell phone communication during the WelTel Kenya1 antiretroviral adherence trial. PLoS One. 2012;7(9):e46033 doi: 10.1371/journal.pone.0046033 23049928 10.1371/journal.pone.0046033 PMC3457960

[25] Crankshaw T, Corless IB, Giddy J, Nicholas PK, Eichbaum Q, Butler LM. Exploring the patterns of use and the feasibility of using cellular phones for clinic appointment reminders and adherence messages in an antiretroviral treatment clinic, Durban, South Africa. AIDS Patient Care STDS. Mary Ann Liebert, Inc. 140 Huguenot Street, 3rd Floor New Rochelle, NY 10801 USA; 2010;24(11):729–34. doi: 10.1089/apc.2010.0146 21039181 10.1089/apc.2010.0146

[26] Martinez M, Salata C, Tucker D, Tanaka D. The Use of Cell Phone Reminder Calls for Assisting HIV-Infected Adolescents and Young Adults to Adhere to Highly Active Antiretroviral Therapy: A Pilot Study. 2006;20(6):1–3. 10.1089/apc.2006.20.438 16789857

[27] Tucker JA, Simpson CA, Roth DL, Stewart KE. Utility of an Interactive Voice Response System to Assess Antiretroviral Pharmacotherapy Adherence Among Substance Users Living with HIV / AIDS in the Rural South. 2013;27(5):280–6. doi: 10.1089/apc.2012.0322 23651105 10.1089/apc.2012.0322 PMC3651686

[28] National AIDS Council (CNCS). Global AIDS Response Progress Report [Internet]. 2012 [cited 2016 Jul 7]. Available from: http://www.unaids.org/en/dataanalysis/knowyourresponse/countryprogressreports/2012countries/ce_MZ_Narrative_Report%255B1%255D.pdf

[29] Jennings L, Ong’ech J, Simiyu R, Sirengo M, Kassaye S, Townsend C, et al Exploring the use of mobile phone technology for the enhancement of the prevention of mother-to-child transmission of HIV program in Nyanza, Kenya: a qualitative study. BMC Public Heal 2013 131. BioMed Central; 2013;13(1):973–81. 10.1186/1471-2458-13-1131 PMC4234194 24308409

[30] Lester RT, Ritvo P, Mills EJ, Kariri A, Karanja S, Chung MH, et al Effects of a mobile phone short message service on antiretroviral treatment adherence in Kenya (WelTel Kenya1): a randomised trial. Lancet. 2010;376(9755):1838–45. doi: 10.1016/S0140-6736(10)61997-6 21071074 10.1016/S0140-6736(10)61997-6

[31] Kop ML Van Der, Memetovic J, Smillie K, Coleman J, Hajek J, Borek N Van, et al Use of the Weltel mobile health intervention at a tuberculosis clinic in British Columbia: a pilot study. J MTM. 2013;2(3).

[32] Johnson BT, Redding CA, DiClemente RJ, Mustanski BS, Dodge B, Sheeran P, et al A network-individual-resource model for HIV prevention. AIDS Behav. 2010;14(0 2):204–21. 20862606 10.1007/s10461-010-9803-z PMC4361779

[33] Brion J. The Patient-Provider Relationship as Experienced by a Diverse Sample of Highly Adherent HIV-Infected People. J Assoc Nurses AIDS Care. Elsevier Ltd; 2014;25(2):123–34. doi: 10.1016/j.jana.2013.01.006 23809659 10.1016/j.jana.2013.01.006

[34] Russell J, Krantz S, Neville S. The patient-provider relationship and adherence to highly active antiretroviral therapy. J Assoc Nurses AIDS Care. 2004;15(5):40–7. doi: 10.1177/1055329004269283 15358924 10.1177/1055329004269283

[35] Hoffman JA, Cunningham JR, Suleh AJ, Sundsmo A, Dekker D, Vago F, et al Mobile direct observation treatment for tuberculosis patients: a technical feasibility pilot using mobile phones in Nairobi, Kenya. Am J Prev Med. 2010;39(1):78–80. doi: 10.1016/j.amepre.2010.02.018 20537846 10.1016/j.amepre.2010.02.018

