# Supplementary material for: Mobile health treatment support intervention for HIV and tuberculosis in Mozambique: Perspectives of patients and healthcare workers
Source: PLoS One. 2017 Apr 18;12(4):e0176051. doi: 10.1371/journal.pone.0176051 (PMC5395223; doi:10.1371/journal.pone.0176051)
Supplement: S1 Appendix — (DOCX) [file pone.0176051.s001.docx]

**S1 Appendix 1. Questionnaire for health care workers**

1. Position / Role at health facility:

Nurse  Medical doctor  Health care technician  Pharmacist

Health Counsellor  Other: _________

1. What is your gender?

Man  Female

1. What is your age?

*Read the following statements and choose/mark one of the options below each statement:* **strongly disagree/disagree/neither agree nor disagree/agree/strongly agree**

1. The SMS system can help to reduce the number of patients who miss appointments.
2. The SMS system can help to reduce the number of patients who miss collecting medication.
3. The SMS system helps me to discuss health issues related to the patient.
4. There are risks with SMS system.
5. If you agreed, indicate some.
6. There are benefits of motivational and / or education messages.
7. I feel confident with the SMS system.
8. I would recommend other health centers to use the SMS system.
9. If you agreed, explain why.
10. Any idea to contribute for improvement of the SMS system?
