# Supplementary material for: Mobile health treatment support intervention for HIV and tuberculosis in Mozambique: Perspectives of patients and healthcare workers
Source: PLoS One. 2017 Apr 18;12(4):e0176051. doi: 10.1371/journal.pone.0176051 (PMC5395223; doi:10.1371/journal.pone.0176051)
Supplement: S2 Appendix — (DOCX) [file pone.0176051.s002.docx]

**S2 Appendix. Interview guide for patients**

1. Name of the health centre:

Machava 2  Matola 1  Matola 2  Namaacha  Ndlavela

1. What is your gender?

Man  Female

1. What is your age?
2. What is your marital status?

Single  Married   Union of facts  Divorced  Separated  Widower

1. Have children under 10 years under your care?

Yes  No

1. What is your occupation / profession

Unemployed  Student  Employee  Dealer  Other:__________

1. What is your monthly household income in MT?

Less than 2,500 Mt  2500-5000 Mt  5000-7500 Mt  Mt 7500 or more  Dependent parents / family

1. What kind of transportation do you use to go to the health centre?

Public transport   Car (own)   Bike   Walk

1. How long it takes for you to go to the health centre?

Less than 5 min  5-30 min  30-60 min  1-2 hours  More than 2 hours

1. How much you spend per month (on average) for transport to go to the health centre?

Less than 200 Mt  200-500 Mt  500 or more  I don’t know

*Read the following statements and choose/mark one of the options below each statement:* **strongly disagree/disagree/neither agree nor disagree/agree/strongly agree**

1. There are risks with SMS system.
2. If you agreed, indicate some.
3. I feel confident with the SMS system.
4. If not you agreed, why?
5. The SMS system helped me not missing appointments.
6. If you've missed appointments, how many times?

0 - No  1-5  6 - 10  11-15  More than 15

1. If you already missed appointments, what was the main reason?
2. The SMS system helped me to remember to collect medication.
3. If you ever missed to collect medication, how many times?

0 - No  1-5  6 - 10  11-15  More than 15

1. If you ever missed to collect medication, what was/were the main reason(s)?
2. How would you rate the ease of use of the SMS system?

Very difficult  Difficult  Neither difficult nor easy  Easy to use

Very easy to use

1. I think there are benefits of SMS in terms of receiving education and motivational messages.
2. The content of the text messages is very easy to read and understand.
3. There is some content that you think is missing in the SMS system?

Yes  No

1. If answer is yes, please, what content is missing?
2. I am willing to use the SMS system in the future to help me with the same disease or another.
3. I would recommend to other patients using SMS system.
