# Supplementary material for: Mobile health treatment support intervention for HIV and tuberculosis in Mozambique: Perspectives of patients and healthcare workers
Source: PLoS One. 2017 Apr 18;12(4):e0176051. doi: 10.1371/journal.pone.0176051 (PMC5395223; doi:10.1371/journal.pone.0176051)
Supplement: S1 Table — (DOCX) [file pone.0176051.s003.docx]

**S1 Table. Demographic characteristics of patients**

| **Variable** | | **Frequency** | **%** |
| --- | --- | --- | --- |
| **Intervention treatment group (n=141)** | |  |  |
|  | HIV | 72 | 51 |
|  | TB-HIV | 69 | 49 |
| **Gender (n=141)** | | | |
|  | Female | 82 | 58 |
|  | Male | 59 | 42 |
| **Age (median, Q1 – Q3)** | | 38.0 (34 – 45) |  |
| **Marital status (n=139)** | |  |  |
|  | Single | 67 | 48 |
|  | In relationship | 72 | 52 |
| **Has children under 10 years in their household (n=121)** | | | |
|  | Yes | 62 | 51 |
|  | No | 59 | 4 |
| **Occupation (n=120)** | |  |  |
|  | Unemployed | 14 | 12 |
|  | Self-employed | 18 | 15 |
|  | Employed | 88 | 73 |
| **Average monthly household income in USD (n=121)** | | | |
|  | < 78 | 34 | 28 |
|  | 78 – 156 | 46 | 38 |
|  | 156 - 234 | 16 | 13 |
|  | >= 234 | 10 | 8 |
|  | Dependent of parents/family | 15 | 13 |
| **Mode of transportation to the healthcare centre (n=122)** | | | |
|  | Public transport | 86 | 70 |
|  | Private transport | 4 | 3 |
|  | Bicycle | 2 | 2 |
|  | Walking | 30 | 25 |
| **Time to the health centre in minutes (n=122)** | | | |
|  | Less than 5 | 4 | 3 |
|  | 5 - 29 | 59 | 49 |
|  | 30 - 59 | 34 | 28 |
|  | 60 - 120 | 21 | 17 |
|  | >= 120 | 4 | 3 |
| **Average monthly spent on transportation in USD (n=122)** | | | |
|  | < 6.25 | 59 | 48 |
|  | 6.25 – 15.63 | 18 | 15 |
|  | >= 15.63 | 5 | 4 |
|  | Didn't know | 40 | 33 |
