# Supplementary material for: Mobile health treatment support intervention for HIV and tuberculosis in Mozambique: Perspectives of patients and healthcare workers
Source: PLoS One. 2017 Apr 18;12(4):e0176051. doi: 10.1371/journal.pone.0176051 (PMC5395223; doi:10.1371/journal.pone.0176051)
Supplement: S2 Table — (DOCX) [file pone.0176051.s004.docx]

**S2 Table. Summary of patients’ responses**

|  | Responses^a^ | | | | | | | | | |
| --- | --- | --- | --- | --- | --- | --- | --- | --- | --- | --- |
|  | 1  strongly disagree | | 2 | | 3 | | 4 | | 5  strongly agree | |
| Item | HIV | TB | HIV | TB | HIV | TB | HIV | TB | HIV | TB |
| 1. I think there are risks with the SMS system | 60 | 26 | 0 | 33 | 3 | 3 | 0 | 4 | 4 | 2 |
| 2. I feel confident with the SMS system | 1 | 0 | 1 | 2 | 0 | 4 | 2 | 9 | 64 | 53 |
| 3. The SMS system helped me not to miss appointments | 0 | 0 | 0 | 0 | 1 | 1 | 6 | 4 | 61 | 63 |
| 4. The SMS system helped me not to miss to collect medications | 0 | 0 | 0 | 0 | 7 | 1 | 0 | 5 | 61 | 62 |
| 5. I found the content of SMS text messages to be very easy to read and understand | 0 | 0 | 1 | 1 | 1 | 3 | 6 | 16 | 60 | 47 |
| 6. I am willing to use the SMS system in the future to help me manage the same or other disease | 0 | 0 | 0 | 0 | 0 | 2 | 2 | 2 | 65 | 64 |
| 7. I would recommend other patients to use the SMS system | 0 | 0 | 0 | 0 | 0 | 4 | 4 | 3 | 63 | 61 |
| 8. I think there are benefits of the SMS system in terms of receiving education and motivational messages | 0 | 0 | 2 | 5 | 0 | 0 | 4 | 2 | 59 | 61 |

^a^Response options: 1 = strongly disagree, 2 = disagree, 3 = neutral, 4 = agree, 5 = strongly agree.
